# An integrated assessment of histopathological changes of the enteric neuromuscular compartment in experimental colitis

**DOI:** 10.1111/jcmm.12428

**Published:** 2014-12-17

**Authors:** Chiara Ippolito, Cristina Segnani, Mariella Errede, Daniela Virgintino, Rocchina Colucci, Matteo Fornai, Luca Antonioli, Corrado Blandizzi, Amelio Dolfi, Nunzia Bernardini

**Affiliations:** a Unit of Histology, Department of Clinical and Experimental Medicine, University of PisaPisa, Italy; b Department of Basic Medical Sciences, Neurosciences and Sensory Organs, University of Bari School of MedicineBari, Italy; c Division of Pharmacology, Department of Clinical and Experimental Medicine, University of PisaPisa, Italy

**Keywords:** colonic inflammatory fibrosis, DNBS, wall remodelling, neuromuscular compartment, myenteric ganglia

## Abstract

Bowel inflammatory fibrosis has been largely investigated, but an integrated assessment of remodelling in inflamed colon is lacking. This study evaluated tissue and cellular changes occurring in colonic wall upon induction of colitis, with a focus on neuromuscular compartment. Colitis was elicited in rats by 2,4-dinitrobenzenesulfonic acid (DNBS). After 6 and 21 days, the following parameters were assessed on paraffin sections from colonic samples: tissue injury and inflammatory infiltration by histology; collagen and elastic fibres by histochemistry; HuC/D, glial fibrillar acidic protein (GFAP), proliferating cell nuclear antigen (PCNA), nestin, substance P (SP), von Willebrand factor, c-Kit and transmembrane 16A/Anoctamin1 (TMEM16A/ANO1) by immunohistochemistry. TMEM16A/ANO1 was also examined in isolated colonic smooth muscle cells (ICSMCs). On day 6, inflammatory alterations and fibrosis were present in DNBS-treated rats; colonic wall thickening and fibrotic remodelling were evident on day 21. Colitis was associated with both an increase in collagen fibres and a decrease in elastic fibres. Moreover, the neuromuscular compartment of inflamed colon displayed a significant decrease in neuron density and increase in GFAP/PCNA-positive glia of myenteric ganglia, enhanced expression of neural SP, blood vessel remodelling, reduced c-Kit- and TMEM16A/ANO1-positive interstitial cells of Cajal (ICCs), as well as an increase in TMEM16A/ANO1 expression in muscle tissues and ICSMCs. The present findings provide an integrated view of the inflammatory and fibrotic processes occurring in the colonic neuromuscular compartment of rats with DNBS-induced colitis. These morphological alterations may represent a suitable basis for understanding early pathophysiological events related to bowel inflammatory fibrosis.

## Introduction

Inflammatory bowel diseases (IBDs) are complex pathological conditions, which must be considered more than mere inflammatory reactions. Indeed, because of their relapsing bouts and chronic course, both Crohn's disease and ulcerative colitis (UC) can progress to fibrosis, resulting in pharmacologically unmanageable alterations, which can be resolved by disabling surgical resections 1,2. Although Crohn's disease is described as the IBD with the most fibrogenic trend, an involvement of the whole intestinal wall can be appreciated also in UC. Besides mucosal and submucosal lesions, UC patients display also abnormalities of neuromuscular compartment, with alterations of myenteric neuron/glia index and interstitial cells of Cajal (ICCs) 3–5. Together with these abnormalities, the occurrence of fibrotic rearrangements in the colon of UC patients 6,7 may represent the consequence of a neglected problem, which may worsen the picture of clinical symptoms 8.

Integrated data on the pathophysiology of inflammatory and fibrotic processes associated with human colitis are very scarce 1,9. Thus, suitable pre-clinical models of colitis, reflecting the histopathological features of human disease, can be useful for investigating the pathogenic mechanisms leading to colitis and, thereby, to wall remodelling 2. Studies evaluating concomitantly both inflammatory injuries and fibrotic rearrangements throughout the colonic wall in the setting of colitis are also lacking. Indeed, a wide range of morpho-functional parameters has been previously characterized in experimental colitis, but, in most cases, single parameters (*e.g*. either extracellular matrix deposition, inflammatory indexes, or myenteric neuron density) were examined with different methods (*e.g*. histochemistry, immunofluorescence, molecular biology techniques), in different samples (*e.g*. tissue sections, cultured cells), by different authors and at different time-points. In this regard, besides the typical histopathological lesions and collagen infiltration described in several studies 10–15, scarce attention has been paid to rearrangements occurring in the neuromuscular compartment, even though morphological alterations in this district may account for enteric dysmotility associated with colonic inflammation.

Based on this background, an integrated assessment of a panel of parameters, aimed at analysing, in the same model and at the same time-points, a number of morphological and molecular changes occurring in the neuromuscular compartment in the setting of colonic inflammation, is expected to provide interesting insights into the pathophysiological relationship between bowel inflammation and fibrotic remodelling. Here, we propose that the model of colitis elicited by 2,4-dinitrobenzenesulfonic (DNBS) in the rat 16, which shares a number of pathological features with human IBDs, can be suitable for investigating tissue remodelling associated with colonic fibrosis, particularly at its early stages. To this aim, colonic samples from DNBS-treated rats were examined for gross morphology and histopathological lesions as well as for molecular and cellular markers within the neuromuscular compartment.

## Materials and methods

### Induction of Colitis

Colitis was induced in male Sprague–Dawley rats (*n* = 20) by intrarectal administration of 30 mg of DNBS 16. The animals were housed and handled in full accordance with the provisions of the European Community Council Directive 86-609. Control rats (*n* = 10) were treated with intrarectal saline. Animals were then evaluated on day 6 and 21 from DNBS administration to assess the development of colonic inflammation and fibrosis. At these time-points, animals were killed and the colon was excised. Colonic inflammation was examined both macroscopically and histologically 16.

### Determination of tissue myeloperoxidase

Myeloperoxidase (MPO) levels in colonic tissues were determined as previously reported 17, and taken as a quantitative index to estimate the degree of mucosal infiltration by polymorphonuclear cells. The results were expressed as ng of MPO per 100 mg of tissue.

### Isolated colonic smooth muscle cells

Rat colonic smooth muscle cells (SMCs) were explanted from *tunica muscularis* 18. Briefly, colonic specimens from control and inflamed animals were excised, washed several times with cold, sterile PBS, and the muscular layers were separated from mucosa and submucosa. The specimens of colonic muscular tissue were then minced and incubated in complete DMEM growth medium (Gibco, Life Technology Italia, Monza, Italy), under 5% CO_2_ at 37°C. Upon confluence, the explants were dissociated by trypsin. Isolated colonic smooth muscle cells (ICSMCs) were then maintained in DMEM 10% foetal bovine serum and used until the fifth passage. Care was taken to verify that ICSMCs displayed and maintained a SMC phenotype by immunostaining for standard markers 19 (data not shown).

### Immunoblotting in colonic tissues and ICSMCs

Colonic specimens were dissected to separate the mucosal/submucosal layer from underlying neuromuscular tissues. Samples of colonic muscular tissue or ICSMCs were homogenized in RIPA lysis buffer (Cole Parmer homogenizer, Generalcontrol SpA, Milano, Italy). Homogenates were spun by centrifugation at 20,000 × g for 15 min. at 4°C. Supernatants were then separated from pellets and stored at −80°C. Protein concentration was determined by the Bradford method (Protein Assay Kit; Bio-Rad, Hercules, CA, USA). Equivalent amounts of protein lysates (50 μg for tissues and 10 μg for ICSMCs) were separated by 8% SDS-PAGE for immunoblotting. After transfer onto a PVDF membrane, the blots were blocked and incubated overnight with a rabbit anti-collagen I antibody (Ab34710; Abcam, Cambridge, UK) or a goat anti-transmembrane 16A/Anoctamin1 (TMEM16A/ANO1) antibody (Table1). After repeated washings with TBS-T, appropriate secondary peroxidase-conjugated antibodies (Santa Cruz Biotech, Santa Cruz, CA, USA) were added for 1 hr at room temperature. Immunoreactive bands were then visualized by incubation with chemiluminescent reagents (Immobilon reagent; Millipore, Billerica, MA, USA), and examined by Kodak Image Station 440 for signal detection. To ensure equal sample loading, blots were stripped and reprobed for determination of β-actin by a specific antibody (P5747; Sigma-Aldrich, Milan, Italy).

**Table 1:** Antibodies used for immunohistochemistry and immunofluorescence

Primary antibodies	Clone	Host	Dilution	Code and source
c-Kit (CD117)	PAb	Rabbit	1:200	PC34; Calbiochem, Darmstadt, Germany
GFAP	PAb	Rabbit	1:100	Z0334; Dakocytomation
HuC/D	MAb	Mouse	1:250	A-21271; Molecular Probes
Nestin	MAb	Mouse	1:400	MAB353; Millipore
PCNA	MAb	Mouse	1:200	M 0879; Dakocytomation
SP	MAb	Rat	1:2000	Sc-21715; Santa Cruz Biotech
TMEM16A/ANO1	PAb	Goat	1:100	Sc-69343; Santa Cruz Biotech
vWF	PAb	Rabbit	1:400	Ab6994; Abcam

Secondary antibodies	Host	Dilution	Code and source
Anti-goat Alexa 568	Donkey	1:300	A-11057; Invitrogen, Eugene, OR, USA
Anti-mouse Alexa 488	Goat	1:300	A-11029; Invitrogen
Anti-rabbit Alexa 568	Goat	1:300	A-11011; Invitrogen
Biotinylated anti-goat IgG	Rabbit	1:200	AP106B; Millipore
Biotinylated anti-mouse IgG*	Goat	1:200	BA-9200; Vector Lab, Burlingame, CA, USA
Biotinylated anti-rabbit IgG	Goat	1:200	BA-1000; Vector Lab

* Revealed by streptavidin-conjugated Alexa 488 for immunofluorescence.

c-Kit (CD117) proto-oncogene, receptor-tyrosine kinase; GFAP, glial fibrillar acid protein; HuC/D, human neuronal proteins C and D; MAb, monoclonal antibody; PAb, polyclonal antibody; PCNA, proliferating cell nuclear antigen; SP, Substance P; TMEM16A/ANO1, transmembrane16A/anoctamin1; vWF, von Willebrand factor.

### Histology, histochemistry, immunohistochemistry and confocal microscopy

Formalin-fixed full-thickness samples were serially cross-sectioned (10 μm-thick). Sections were processed for haematoxylin/eosin staining, histochemical staining for collagen and elastic fibres, and immunostaining with various antibodies and reagents (Table1). To measure the thickness of *tunica muscularis,* a morphometric analysis was carried out from images captured with 20× objective using the Image Analysis System ‘L.A.S. software v.4’ (Leica Microsystems, Cambridge, UK). Tissue collagen deposition was evaluated by histochemical staining with Sirius Red and Fast Green in saturated picric acid solution 11: collagen fibres (red) and cellular, non-collagen proteins (green) were quantitatively estimated within the respective colonic area (whole wall or *tunica muscularis*). Elastic fibres were stained in black with orcein in 70% ethanol acidic solution.

To perform immunohistochemistry, sections were processed as previously described 5 and incubated overnight at 4°C with primary antibodies, with the purpose of detecting myenteric neurons and glia, ICCs, proliferating cells and new vessels (Table1). Slides were then exposed to biotinylated immunoglobulins, peroxidase-labelled streptavidin complex and 3.3′-diaminobenzidine tetrahydrochloride (DakoCytomation, Glostrup, Denmark). Sections were examined by a Leica DMRB light microscope, and representative photomicrographs were taken by a DFC480 digital camera (Leica Microsystems).

Double immunofluorescence was carried out with primary antibodies against glial fibrillar acidic protein (GFAP) combined with proliferating cell nuclear antigen (PCNA) or nestin, and von Willebrand Factor (vWF) combined with nestin 20. Briefly, sections were sequentially incubated with: 0.5% Triton X-100 (Merck KGaA, Darmstadt, Germany) in PBS solution, Protein Block Serum Free (Dako Cytomation, Glostrup, Denmark); combined primary antibodies (overnight at 4°C), revealed by appropriate fluorophore-conjugated secondary antibody or biotinylated secondary antibody followed by fluorophore-conjugated streptavidin; nuclear counterstaining with TO-PRO3 (Molecular Probes, Eugene, OR, USA). Stainings were examined under a Leica TCS SP5 confocal laser-scanning microscope (Leica Microsystems, Mannheim, Germany) using a sequential scan procedure. Confocal images were taken at 250–500 nm intervals through the z-axis of sections, by means of 40× and 63× oil lenses. Z-stacks of serial optical planes were analysed by confocal software (Multicolor Package; Leica Microsystems).

Negative controls were obtained by omitting primary antibodies. Tissue positive (liver) and negative (cartilage) controls were employed to validate the specificity of TMEM16A/ANO1 antibodies (Fig.2).

### Image analysis and statistics

Histochemical and immunohistochemical findings were quantitatively estimated by two blind investigators 5. Briefly, 5 randomly selected microscopic fields from 3 non-adjacent sections were analysed for each animal (*n* = 6–8) and evaluated by the Image Analysis System ‘L.A.S. software v.4’. Positive areas were expressed as percentage of the total tissue area examined (percentage positive pixels, PPP). Neuronal density was estimated as neuron number within ganglionic area by HuC/D immunostaining of nuclei and/or perikarya 21. To quantify the proliferation of glial cells, the percentage of GFAP-positive glia which displayed PCNA-labelled nuclei was determined 22. Data are given as mean ± SD. Student's *t*-test for unpaired data (two-tailed) was performed to assess statistical differences between groups. A *P* ≤ 0.05 was considered significant. For histochemistry and immunohistochemistry, quantitative variations were expressed as fold changes, which were calculated as the ratio of the final value over the initial value. For immunoblotting assays, quantitative determination of band intensity was calculated as the ratio between the protein of interest and β-actin.

## Results

### Evaluation of colonic inflammation

At day 6 after DNBS administration, there was a significant decrease in bodyweight, and increments of spleen weight and bowel inflammatory parameters (macroscopic damage and MPO levels). Likewise, at day 21, the bodyweight gain was lower, while systemic and tissue inflammatory parameters were significantly increased in comparison with controls, although MPO drifted towards a recovery as compared with DNBS at day 6 (Table2).

**Table 2:** Systemic and tissue inflammatory parameters in colonic tissue samples collected from rats treated with DNBS or vehicle at day 6 or 21

	Weight variation (%)	Spleen weight (%)	Macroscopic damage score	MPO (ng/100 mg tissue)
Control day 6	+16 ± 2.2	100 ± 2.5	1.4 ± 0.3	6.4 ± 1.8
DNBS day 6	−7 ± 1.8*	128 ± 4*	8.5 ± 1.2*	28.8 ± 5.2*
Control day 21	+38 ± 3	100 ± 4.5	1.3 ± 0.2	5.2 ± 2
DNBS day 21	+12 ± 1.4*^,^†	138 ± 3*	7.2 ± 1.1*	16.4 ± 3.8*^,^†

* *P* < 0.05 significant difference *versus* the respective group treated with vehicle.

† *P* < 0.05 significant difference *versus* DNBS day 6.

MPO*:* myeloperoxidase.

### Histology

**Fig 1:**
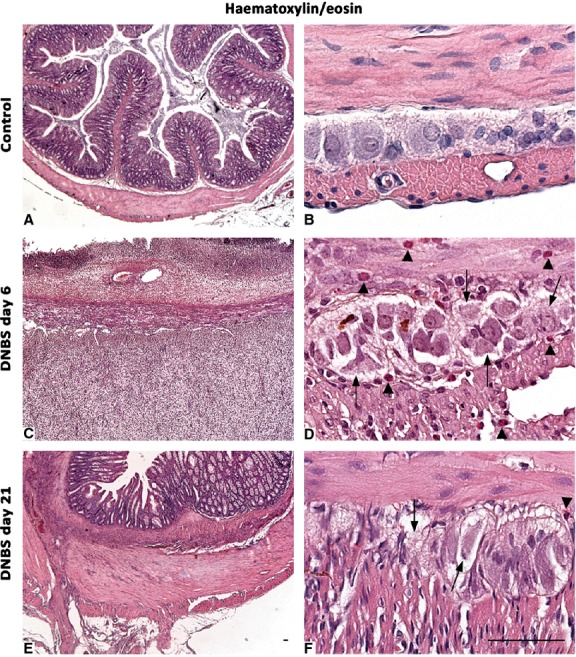
Histological appearance of haematoxylin/eosin-stained full-thickness colonic samples in control rats (A and B), or animals with DNBS-induced colitis at day 6 (C and D) and day 21 (E and F). The colonic wall of controls shows normal morphological features (A), with compact myenteric ganglia, which are plenty of neurons and glial cells (B). Colonic specimens from rats with colitis are damaged and thickened (C and E): myenteric ganglia appear to be vacuolized, with altered cells (arrows), and infiltrated by eosinophil granulocytes (D and F arrowheads), which are widely present also throughout the *tunica muscularis*; scale bars = 50 μm.

Colonic samples from controls displayed a normal tissue architecture, with myenteric ganglia filled of neurons and glial cells (Fig.1). At day 6 after DNBS, transmural lesions, consistent with colitis, were detected: ulcerated mucosa, infiltrated *tunica submucosa*, thickened *tunica muscularis*, which increased by 3.4-fold (282.00 ± 0.49) *versus* control (83.65 ± 0.23, *P* ≤ 0.001), along with infiltrations with macrophages, neutrophils and eosinophils. Myenteric ganglia were infiltrated by eosinophils, and displayed cell loss and lacunar spaces (Fig.1). At day 21, the colonic wall was still thickened, with a 3.6-fold increase of *tunica muscularis* (300.95 ± 0.87, *P* ≤ 0.001) *versus* controls, and affected by residual leucocyte infiltration, which consisted mainly of eosinophils. Myenteric ganglia still displayed appreciable alterations (vacuoles and eosinophils) (Fig.1).

### Tissue distribution of collagen and elastin

**Fig 2:**
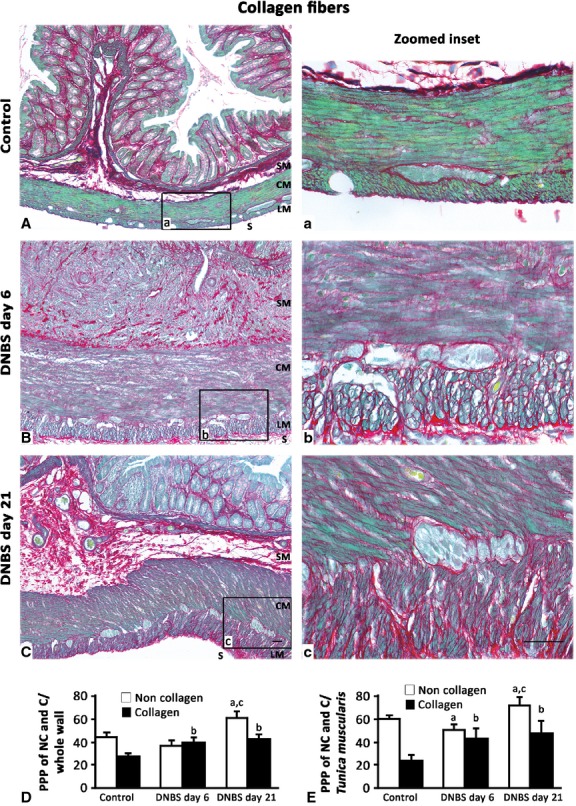
Representative photomicrographs of full-thickness colon showing the distribution pattern of Sirius Red-stained collagen fibres and Fast Green-stained non-collagen proteins in control rats (A, a inset) or animals with DNBS-induced colitis at day 6 (B, b inset) and day 21 (C, c inset). Collagen deposition increases at day 6 and day 21, as compared with controls. (SM, submucosal layer; CM and LM, circular and longitudinal muscle respectively; S, serosa); scale bars = 50 μm. Quantitative estimations of collagen and non-collagen proteins were performed by image analysis and expressed as percentage of positive pixels (PPP) calculated on the whole colonic wall (D) or *tunica muscularis* (E) tissue area examined. Column graphs display the mean values of PPP ± SD obtained from eight rats. ^a,b^*P* ≤ 0.05 *versus* respective controls, ^c^*P* ≤ 0.05 *versus*DNBS day 6.

**Fig 3:**
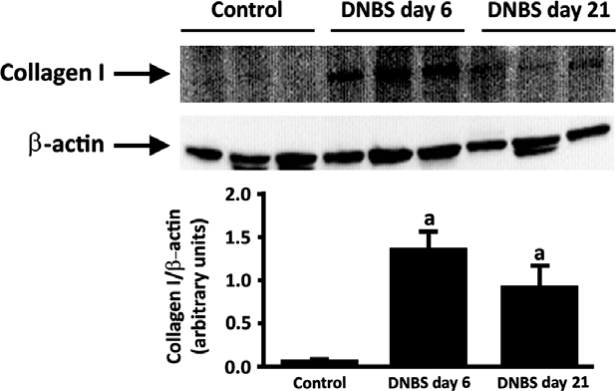
Western blot analysis of collagen I in the colonic neuromuscular layer of control and DNBS-treated rats. Tissue specimens were obtained from control rats as well as animals with colitis after 6 days (DNBS day 6) or 21 days (DNBS day 21) from treatment with DNBS. The column graph displays mean values of densitometric analysis ±SD obtained from six animals. ^a^*P* ≤ 0.05 *versus* controls.

The distribution pattern of collagen underwent considerable changes in DNBS-treated animals (Fig.2). Control colon displayed transmural Sirius Red-stained collagen proteins. After DNBS administration, collagen deposition within the whole colonic wall increased significantly at both day 6 and day 21 (Fig.2), displaying a scattered distribution within *tunica submucosa* and *muscularis*. When the analysis was focused on *tunica muscularis*, collagen fibres were found to be arranged into thick bundles, mainly along myenteric ridge and longitudinal layer, and significantly increased in comparison with controls (Fig.2E). These increments were consistent with data from immunoblot analysis of collagen I (Fig.3).

**Fig 4:**
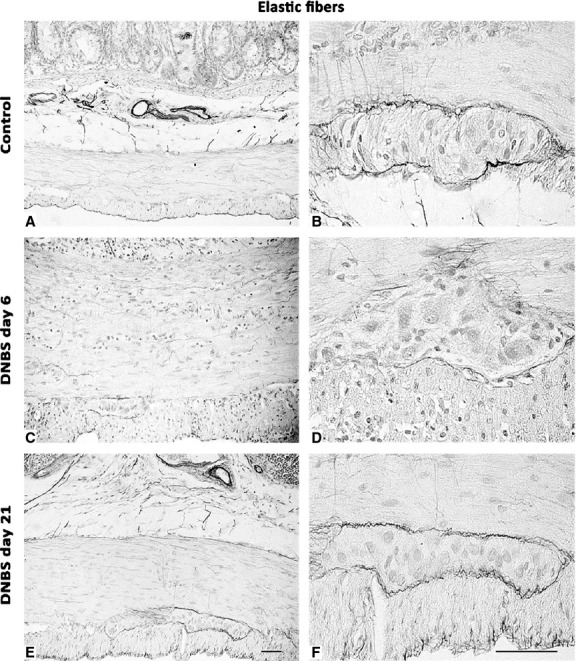
Representative photomicrographs of full-thickness colon displaying the distribution pattern of orcein-stained elastic fibres in control rats (A and B) or animals with DNBS-induced colitis at day 6 (C and D) and day 21 (E and F). Elastic fibres decrease, mainly along the myenteric ridge, by day 6, and return to a normal pattern by day 21; scale bars = 50 μm.

The distribution pattern of elastic fibres (Fig.4), which were detected throughout the whole thickness of control colon (4.09 ± 1.68; *n* = 6), with a predominance in vessel walls, was markedly altered in animals with DNBS-induced colitis. In particular, at day 6, elastic fibres were decreased by 4.5-fold (0.91 ± 0.25; *P* ≤ 0.05; *n* = 6), while they recovered at day 21 (3.81 ± 0.67; *n* = 6).

### Immunostaining

#### HuC/D, GFAP and PCNA

**Fig 5:**
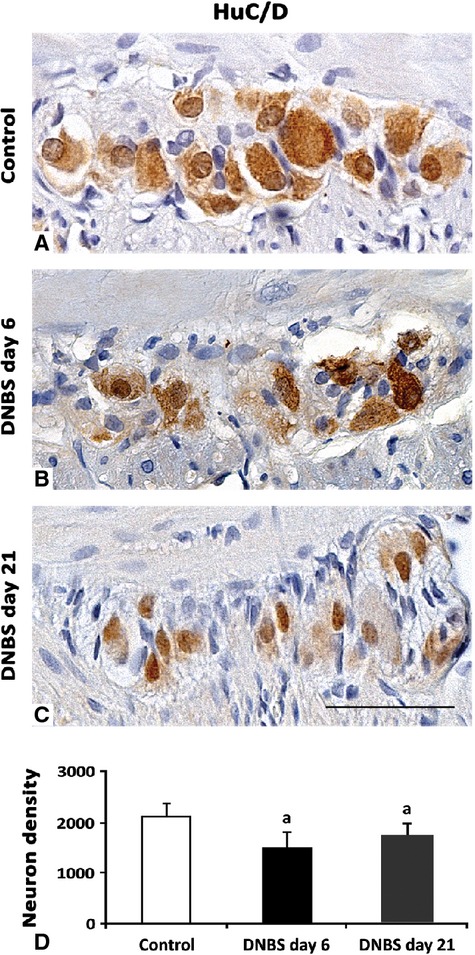
HuC/D-immunostained myenteric ganglia in cross-sections of rat colonic specimens. In normal ganglia, neurons are abundant and markedly HuC/D immunoreactive (A). At day 6 and 21 from DNBS administration, there is a decrease in both neuron density and their immunoreactivity (B and C respectively), even if at day 21 the morphology of ganglia is more similar to that of controls (C); scale bar = 50 μm. (D) The column graph displays mean values of neuron density (neurons/mm^2^) ±SD obtained from six rats. ^a^*P* ≤ 0.05 *versus* controls.

Enteric neurons were detected by cytoplasmic and/or nuclear HuC/D immunostaining, as observed in control colon (Fig.5). At day 6, myenteric neurons displayed an inhomogeneous HuC/D staining and several cytoplasmic vacuoles, whereas at day 21, neurons appeared smaller than controls, displaying a scant cytoplasmic HuC/D expression. At day 6 and 21, the number of myenteric HuC/D-positive neurons in DNBS-treated rats was reduced by 1.6- and 1.1-fold respectively.

**Fig 6:**
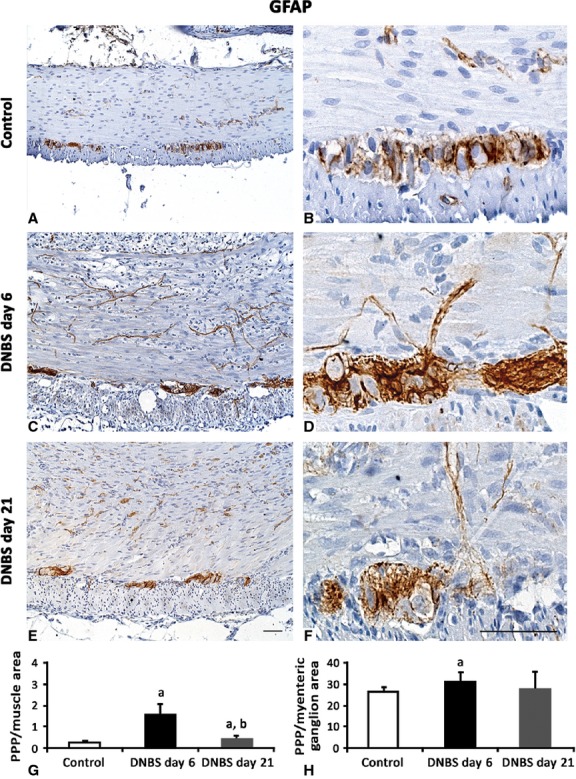
Representative pictures of GFAP immunostaining in colonic *tunica muscularis* and myenteric ganglia from control rats (A and B) or animals with DNBS-induced colitis at day 6 (C and D) and day 21 (E and F). By comparison with controls, at day 6 GFAP expression significantly increases in muscle layers and myenteric ganglia; scale bars = 50 μm. Quantitative estimation of GFAP expression was obtained by image analysis and expressed as percentage of positive pixels (PPP) calculated on the whole *tunica muscularis* (G) or myenteric ganglionic (H) area examined. Column graphs display mean values of PPP ±SD obtained from six rats. ^a^*P* ≤ 0.05 *versus* controls; ^b^*P* ≤ 0.05 *versus*DNBS day 6.

**Fig 7:**
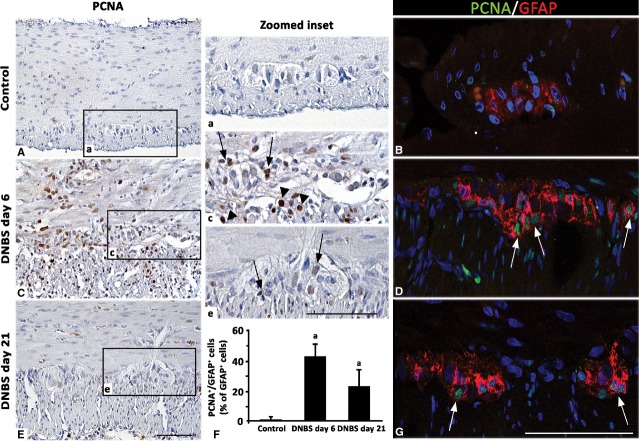
Representative pictures of PCNA immunostaining in colonic *tunica muscularis* and myenteric ganglia from control rats (A) or animals with DNBS-induced colitis at day 6 (C) and day 21 (E). By comparison with controls, on day 6 PCNA positivity is expressed mainly along the myenteric ridge in the nuclei of small ganglionic and muscle cells (arrows and arrowheads respectively), while it decreases on day 21. Confocal microscopy representative images of PCNA/GFAP double immunolabelled sections show GFAP-positive glial cells with PCNA-nuclei at day 6 and 21 (arrows; D and G) compared with ganglia from control rats (B); scale bars = 50 μm. (F) The column graph displays mean values of the percentage of GFAP-positive glial cells with PCNA-labelled nuclei over GFAP-positive glia of myenteric ganglia ±SD obtained from six rats. ^a^*P* ≤ 0.05 *versus* controls.

Glial cells were identified by their reactivity to anti-GFAP immunostaining (Fig.6). At day 6, in DNBS-treated rats, the amount of GFAP staining in inflamed colon increased within the muscle layers (6.7-fold), which appeared rich in fibroblast-like shaped GFAP-positive cells, as well as in myenteric ganglia (1.2-fold). In these ganglia, several GFAP-positive glial cells showed PCNA-positive nuclei (43%), consistent with a glial proliferating feature, which was maintained at day 21 (24%) (Fig.7). At day 21, the GFAP immunostaining value of inflamed colon was 0.44 ± 0.13 *versus* control 0.23 ± 0.09 (*P* < 0.05; *n* = 6) in muscle layers and 27.75 ± 8.11 (*n* = 6) *versus* control 26.32 ± 1.89 (*n* = 6) in myenteric ganglia, thus remaining significantly high only within the *tunica muscularis* (1.9-fold), but not in the ganglionic area (1.0-fold; Fig.6).

#### Nestin, GFAP and vWF

**Fig 8:**
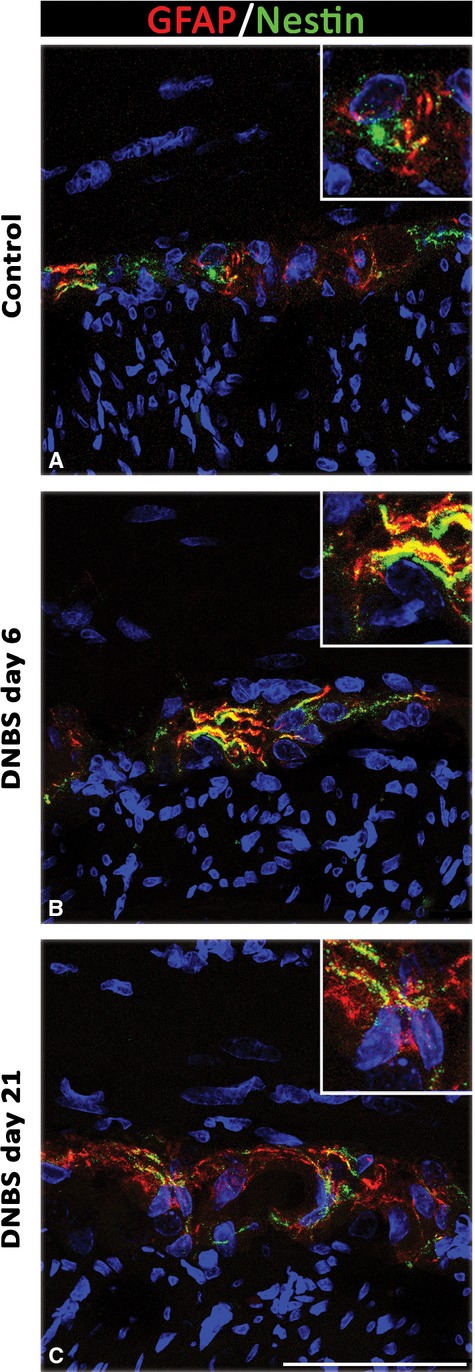
Confocal microscopy representative images of GFAP/nestin double-immunolabelled myenteric ganglia from control rats (A) and animals with DNBS-induced colitis on day 6 and 21 (B and C). In the myenteric ganglia of inflamed rats, glial cells display nestin immunoreactivity, which co-localizes with GFAP in bodies and processes at both day 6 (B, inset) and 21 (C, inset); scale bar = 50 μm.

**Fig 9:**
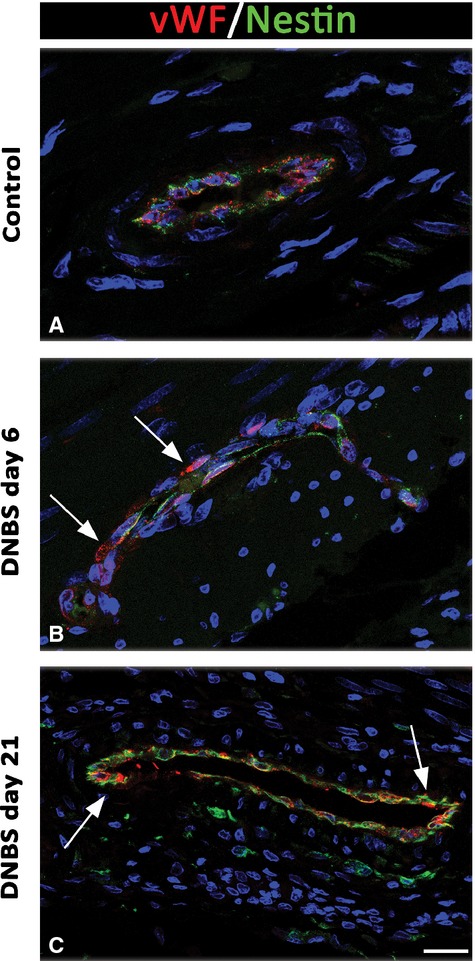
Confocal microscopy representative images of nestin/vWF immunolabelling of *tunica muscularis* from controls and animals with colitis at day 6 and day 21. Inflamed colon (B and C) shows vWF-positive endothelial cells with multiple points of nestin/vWF colocalization on the endothelial profiles (arrows) compared with controls (A); scale bar = 50 μm.

DNBS-treated animals displayed nestin staining in myenteric ganglia, which was co-localized with GFAP, particularly at day 6 (Fig.8). The examination of vessel network, performed by double vWF/nestin immunolabelling, highlighted the presence of newly formed vWF/nestin-positive microvessels in the *tunica muscularis* at day 6 and 21 (Fig.9).

#### Substance P

**Fig 10:**
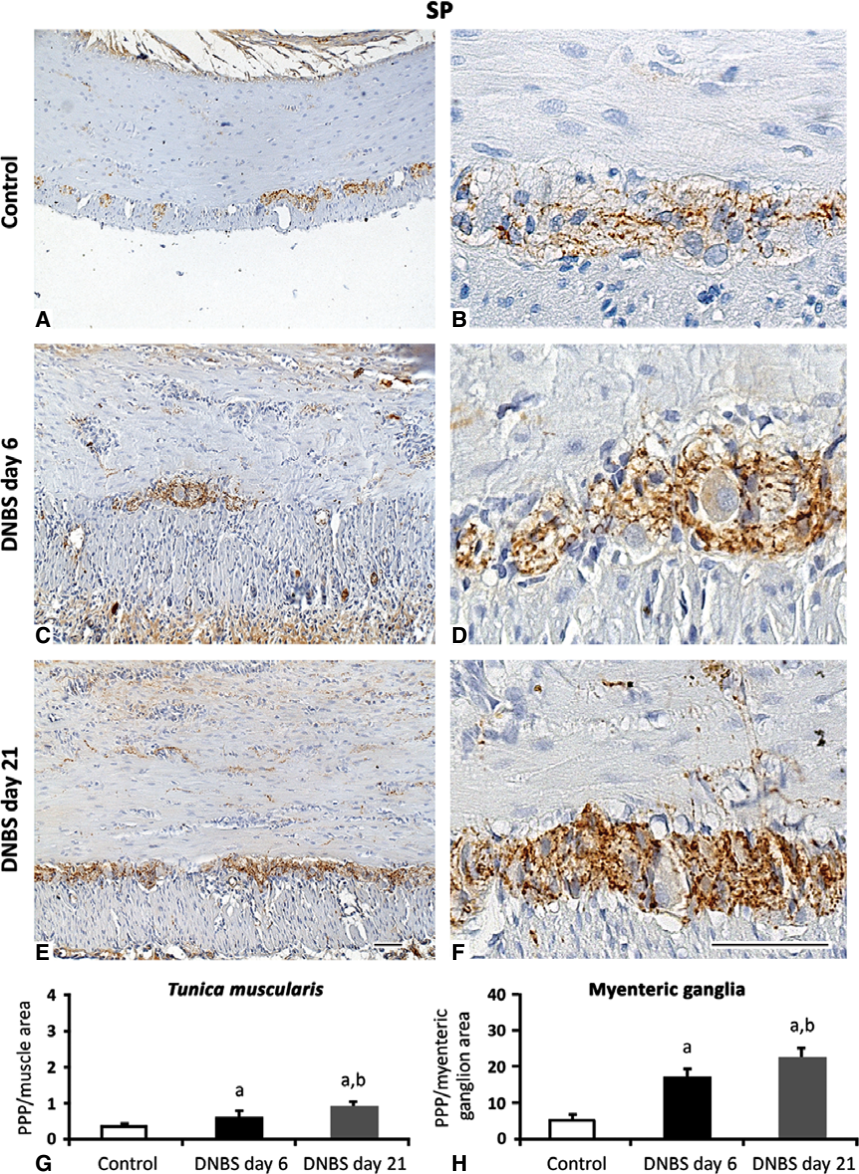
Representative pictures of SP immunostaining in colonic *tunica muscularis* and myenteric ganglia from control rats (A and B) or animals with DNBS-induced colitis at day 6 (C and D) and day 21 (E and F). Note the increase in SP positivity within the muscle layers and myenteric ganglia over the two time-points; scale bars = 50 μm. (G and H) Quantitative estimation of SP expression was obtained by image analysis and expressed as percentage of positive pixels (PPP) calculated on the whole *tunica muscularis* (G) or myenteric ganglionic (H) area examined. Column graphs show mean values of PPP ± SD obtained from six rats. ^a^*P* ≤ 0.05 *versus* controls; ^b^*P* ≤ 0.05 *versus*DNBS day 6.

In control colon, substance P (SP) immunoreactivity was found in nerve fibres of *tunica muscularis* and myenteric ganglia (Fig.10). In the colon from DNBS-treated rats, the density of SP-positive nerve fibres increased both in muscle layers and myenteric ganglia. At day 6 and 21, SP expression in the inflamed colon increased by 1.8- and 2.7-fold in the *tunica muscularis*, and by 3.4- and 4.6-fold in the myenteric ganglia respectively.

#### c-Kit and TMEM16A/ANO1

**Fig 11:**
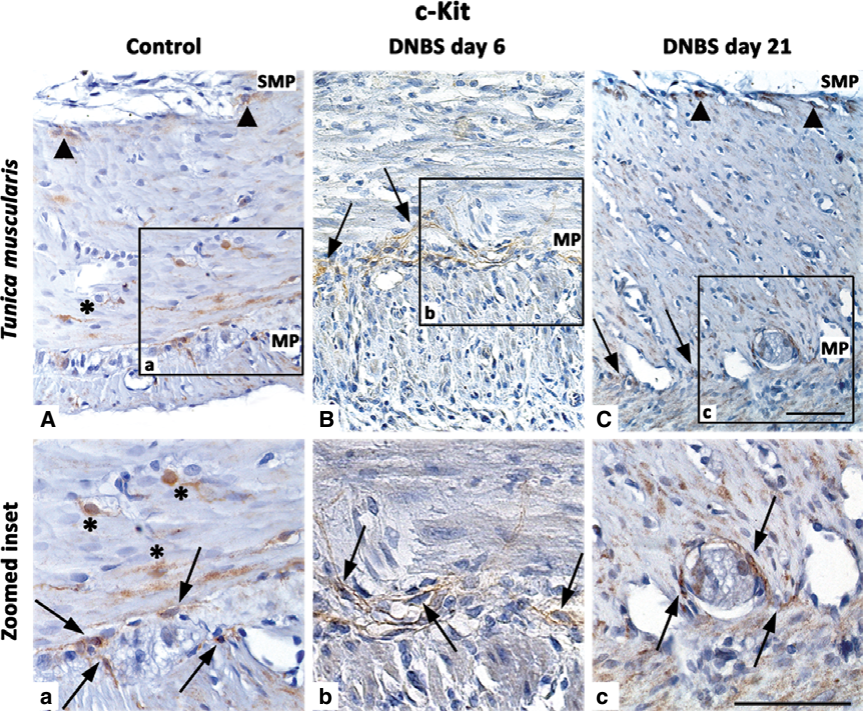
Representative pictures of c-Kit immunostaining in the colonic *tunica muscularis* of control rats (A), or animals with DNBS-induced colitis at day 6 (B) and day 21 (C). Arrowheads, arrows and asterisks highlight ICCs of submucosal *plexus* (SMP), myenteric *plexus* (MP) and intramuscular ICCs respectively. At day 6, a derangement of ICCs is evident, with a partial recovery at day 21; scale bars = 50 μm.

**Fig 12:**
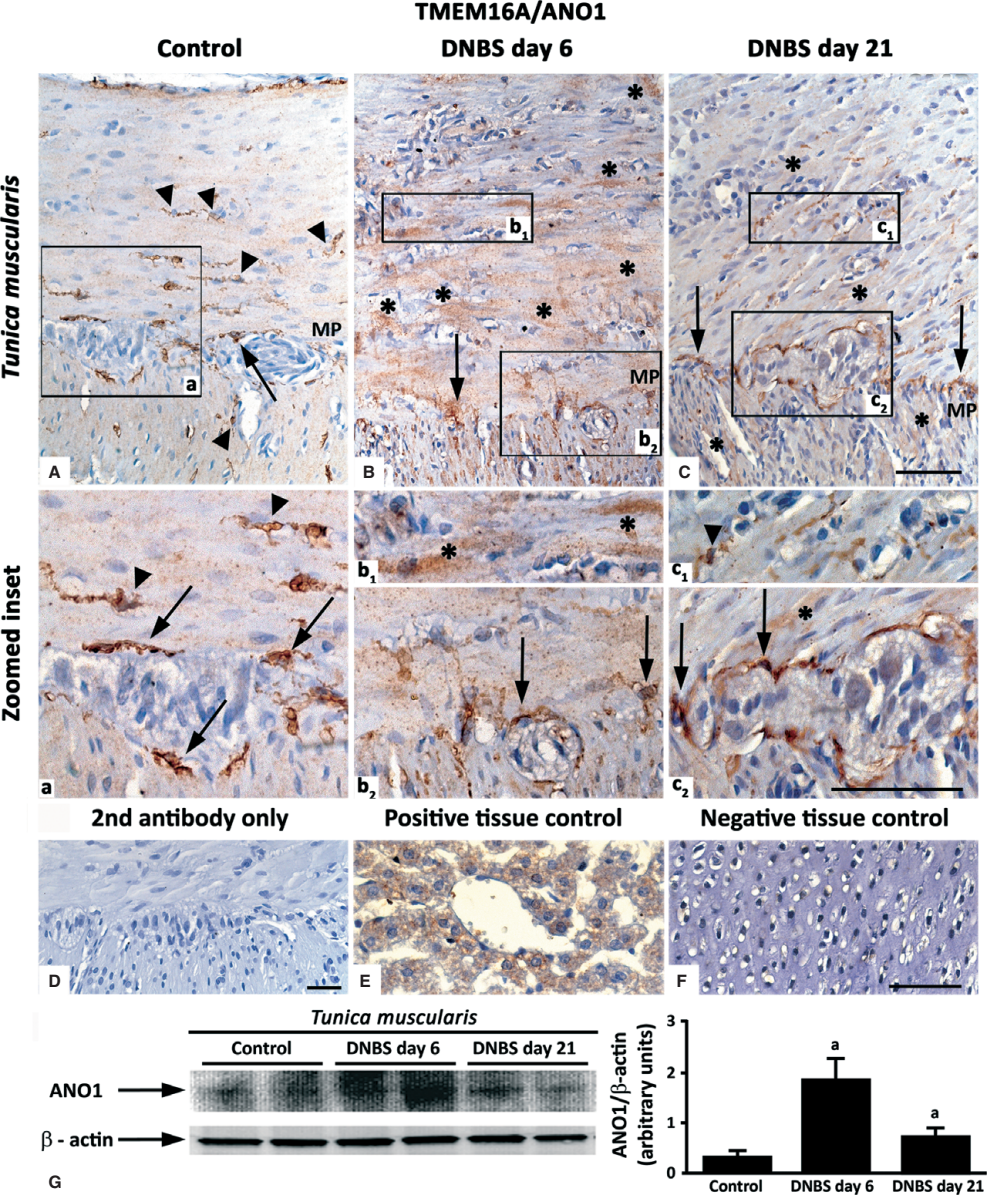
Representative pictures of TMEM16A/ANO1 immunostaining in the colonic *tunica muscularis* of control rats (A) or animals with DNBS-induced colitis at day 6 (B) and day 21 (C). ICCs of myenteric *plexus* (MP) and intramuscular ICCs (arrows and arrowheads respectively) of control colon are immunostained for TMEM16A/ANO1. In the inflamed colon, immunopositive smooth muscle cells are present (B and C; asterisks). Zoomed insets (a, b_1_, b_2_, c_1_, c_2_) on myenteric ridge and circular muscle. Negative control colon (D). Positive (E, liver) and negative (F, cartilage) control tissues; scale bars = 50 μm. (G) Western blot analysis of TMEM16A/ANO1 in the colonic neuromuscular layer of control and DNBS-treated rats. Colonic specimens were obtained from control rats as well as animals with colitis after 6 days (DNBS day 6) or 21 days (DNBS day 21) from treatment with DNBS. The column graph displays mean values of densitometric analysis ±SD obtained from six animals. ^a^*P* ≤ 0.05 *versus* controls.

**Fig 13:**
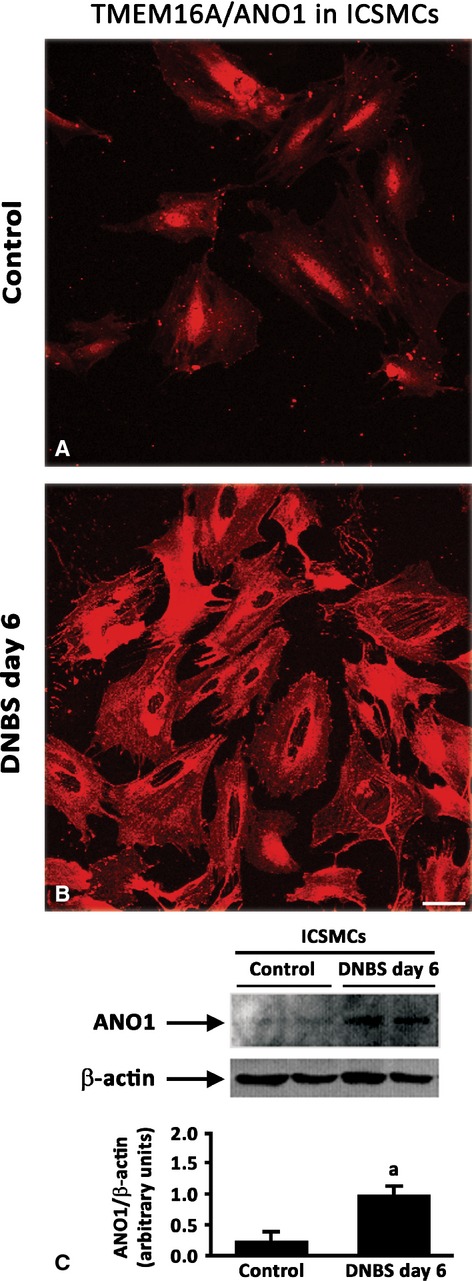
Isolated colonic smooth muscle cells (ICSMCs) obtained from control or inflamed (DNBS day 6) rats (A and B respectively) and immunolabelled for TMEM16A/ANO1; scale bar = 50 μm. (C) Western blot analysis of TMEM16A/ANO1 in ICSMCs from control and DNBS-treated rats (DNBS day 6). The column graph displays mean values of densitometric analysis ±SD obtained from three animals. ^a^*P* ≤ 0.05 *versus* controls.

In control colon, ICCs were identified as spindle-shaped c-Kit-positive and TMEM16A/ANO1-positive cells, endowed with long bipolar processes, mostly running parallel to the axis of SMCs in the *tunica muscularis*, as well as along submucosal and myenteric ridges (Figs11 and 12). In DNBS-treated rats, the morphological features and distribution patterns of ICCs were markedly modified, being maintained only the ICC network around myenteric ganglia. Of note, in inflamed colon, TMEM16A/ANO1 was widely expressed within SMCs of *tunica muscularis*. This finding was confirmed by western blot analysis of colonic tissues, which showed an increased expression of TMEM16A/ANO1 (Fig.12G), and supported further by studies on ICSMCs from rats with colitis at day 6, which displayed high levels of TMEM16A/ANO1 expression (Fig.13).

## Discussion

Cellular and molecular events occurring in colonic inflammation represent an interesting field of investigation, in view of their important implications for related bowel dysfunctions 9. With regard to intestinal dysmotility, which accompanies both inflammatory and fibrotic processes, the pathophysiological bases probably descend from morphological and/or electrophysiological alterations in the neuromuscular compartment, as pointed out by previous reviews 23,24. However, a combined evaluation of both bowel inflammation and fibrosis, with particular regard for the neuromuscular district, is lacking. Therefore, the present study was designed to assess a number of morphological and pathophysiological aspects in the same model of colitis, to obtain an integrated view of changes associated with inflammation and fibrosis, and gain insight into their impact on colonic remodelling. Particular attention was paid to the cellular elements involved in the control of enteric motility.

Although colitis induced by 2,4,6-trinitrobenzenesulfonic acid (TNBS) in mice has been previously employed for investigations on bowel inflammatory fibrosis 1,10,15, we selected the DNBS rat model of colitis for different reasons: (*i*) DNBS is less aggressive than TNBS against the mucosal layer 25; (*ii*) rats develop a maximal degree of colonic inflammation/fibrosis earlier (usually by day 6 26) than mice, which are relatively resistant to the induction of fibrosis (8–12 weeks 27); (*iii*) DNBS-induced inflammatory lesions are consistent with those observed in human IBDs 28.

In our experiments, the wall of inflamed colon from DNBS-treated animals was thickened. At day 6, this picture was concomitant with inflammatory mucosal/submucosal lesions, which were rich in cellular infiltrates (eosinophils, neutrophils), as also documented by the increase in tissue MPO, and a transmural deposition of collagen fibres. These alterations were associated with bodyweight loss and spleen weight increase, in line with previous reports 10,16,29,30. On day 21, besides a decrease in the severity of inflammation, an increased deposition of connective fibres persisted within the *tunica submucosa* and *muscularis*. Accordingly, increased pro-collagen mRNA expression and collagen protein deposition have been reported to occur over the course of fibrotic processes associated with experimental colitis 14,31–33. Furthermore, in our experiments, muscle thickening in the inflamed colon was concomitant with the presence of PCNA-labelled nuclei in the *tunica muscularis* and an increment of non-collagen components, suggesting a hyperplastic reaction of colonic SMCs, in accordance with previous studies in rats with TNBS-induced colitis 10,11. In the present study, colitis was associated also with a loss of elastic fibres, which recovered on day 21. This derangement of collagen/elastin network might well contribute to bowel motor dysfunctions, contributing to weight loss, which is a hallmark of animals with colitis 2,15. Moreover, the decreased density of myenteric HuC/D-positive neurons, as observed in DNBS-treated rats, in accordance with previous studies 34,35, may contribute to colonic motor dysfunctions as well. Of note, such a loss of myenteric neurons appears to occur at an early stage of bowel inflammation, and it is supported by an early activation of pro-apoptotic signals 36, with particular regard for caspase-3 28.

When considering glial cells, the examination of three distinct markers (GFAP, PCNA and nestin) allowed us to observe an activation (GFAP expression increase) and proliferation (PCNA-positive nuclei) of myenteric glial cells in the inflamed colon at day 6 and day 21. Our results expand previous data documenting a glial rearrangement under inflammatory conditions. In particular, activation was observed in cultured rat and human enteric glial cells, which expressed increased GFAP levels, but no increase in proliferation rate, in response to inflammatory cytokines 37,38. By contrast, there was evidence of mitotic activity in myenteric glia under TNBS-induced ileitis 39 and other models of bowel inflammation 40. In addition, the present up-regulation of nestin in activated GFAP-positive myenteric glial cells is consistent with previous observations showing an increased expression of GFAP and nestin in reactive astrocytes of central nervous system under pathological conditions 41,42. Of note, nestin is regarded as a marker of multilineage progenitor cells and an index of potential regenerative activity 43,44. On this basis, the presence of GFAP/PCNA/nestin-positive cells in myenteric ganglia, as observed in our study, suggests a recruitment of activated glial cells with stem potential in the myenteric ganglia of inflamed colon.

In the neuromuscular compartment of inflamed colon, we noted a number of vWF/nestin-positive neovessels and sprouting. Neoangiogenic processes have been described in experimental colitis 35,45,46 and under fibrotic conditions associated with nestin-positive vascular endothelium 47–49. The detection of neovessels during inflammation is of great interest, as they are likely to participate in the initiation of fibrotic remodelling. Indeed, pericytes and endothelium can be regarded as precursors of activated myofibroblasts, and endothelial-to-mesenchymal transition is considered as a relevant step in the pathogenesis of fibrosis 45,46. Highly pertaining to this context are also recent findings, supporting the concept that telocytes, displaying immunoreactivity for platelet-derived growth factor receptor β, contribute to neoangiogenesis in skeletal muscles 50 and might be implicated in vascular fibrogenesis associated with bowel inflammation as well.

The neuromuscular compartment of inflamed colon was found to express increased levels of SP fibres. This increment was most evident in myenteric ganglia, in accordance with previous observations 51–54. SP is involved in the control of enteric motility and intercellular communications between myenteric neurons 55,56. Of interest, glial cells, besides their neurotrophic action, play also neuromodulatory actions on SP neurons, as a result of their ability of increasing neuronal survival and promoting SP expression and release 56. Furthermore, SP is considered as an important factor mediating vasodilation and release of inflammatory mediators, thus regulating the permeability of enteric vessels 53. Interestingly, in human UC elevated levels of SP and its receptors as well as SP-induced leucocyte chemotaxis have been reported 53,57. This panel of activities can qualify SP as a significant factor promoting intestinal fibrogenesis, as suggested also by other studies 51,58.

In the present study, the normal colonic neuromuscular compartment displayed appreciable immunoreactivity for c-Kit and TMEM16A/ANO1. In our hands, TMEM16A/ANO1 was found to be a sensitive marker of ICCs, being expressed in a higher proportion of ICCs in the submucosal and myenteric *plexus*, as compared with c-Kit. However, the distribution pattern of TMEM16A/ANO1 was found to be altered in the inflamed colon. In particular, in parallel with a loss of TMEM16A/ANO1-positive ICCs, we detected TMEM16A/ANO1 in muscle layers and ICSMCs, by both immunostaining and immunoblotting. This finding appears to be original in the context of bowel inflammation, but it is not surprising according to current literature. Indeed, TMEM16A/ANO1 is a Ca^+^-activated chloride channel, currently regarded as a specific marker for ICCs 59. However, it has been reported that other cells equipped with Ca^+^-activated chloride currents may express TMEM16A/ANO1 60–62, which has been also found in SMCs of several organ systems 61,63–67. In addition, elevated levels of TMEM16A/ANO1 staining in SMCs have been reported in models of experimental disease, such as pulmonary hypertension 68 and chronic asthma 69. Thus, considering the wide contribution of TMEM16A/ANO1 to the myogenic activity of vascular SMCs 63,70, its up-regulation in SMCs of inflamed colon, together with the derangement of ICC network, as observed in our study, might contribute to enteric motor dysfunctions associated with bowel inflammation 24. Taken together, these considerations open new pathophysiological and therapeutic perspectives in the field of IBDs. Indeed, therapeutic interventions targeted on Ca^+^-activated chloride channels have been recently proposed for several diseases (*e.g*. activators for cystic fibrosis, and inhibitors for hypertension, asthma, and secretory diarrhoea) 71.

In conclusion, the variety of markers and cells examined in the present study provide an integrated view of the impact of inflammatory and fibrotic processes on colonic neuromuscular compartment. According to our findings, the rat model of DNBS-induced colitis displays significant processes of colonic remodelling, consisting not only in collagen deposition and wall thickening, but also in specific cellular alterations of the neuromuscular units, such as myenteric neurons, glial cells, ICCs and SMCs.
